# Evaluation of age-related changes in translocator protein (TSPO) in human brain using ^11^C-[R]-PK11195 PET

**DOI:** 10.1186/1742-2094-9-232

**Published:** 2012-10-04

**Authors:** Ajay Kumar, Otto Muzik, Varun Shandal, Diane Chugani, Pulak Chakraborty, Harry T Chugani

**Affiliations:** 1 Department of Pediatrics, School of Medicine, Children’s Hospital of Michigan, Detroit Medical Center, Wayne State University, 3901 Beaubien Boulevard, Detroit, MI, USA; 2 Department of Neurology, School of Medicine, Children’s Hospital of Michigan, Detroit Medical Center, Wayne State University, Detroit, MI, USA; 3 Department of Radiology, School of Medicine, Children’s Hospital of Michigan, Detroit Medical Center, Wayne State University, Detroit, MI, USA; 4 Department of Pharmacology, School of Medicine, Children’s Hospital of Michigan, Detroit Medical Center, Wayne State University, Detroit, MI, USA; 5 PET Center, School of Medicine, Children’s Hospital of Michigan, Detroit Medical Center, Wayne State University, Detroit, MI, USA

**Keywords:** Adults, Brain, Children, C-[R]-PK-11195 positron emission tomography, Glial cells, Inflammation, Microglia, Neuroinflammation, PBR, Peripheral benzodiazepine receptor, PK11195 PET, Translocator protein, TSPO

## Abstract

**Background:**

We studied the distribution and expression of translocator protein in the human brain using ^11^C-[R]-PK-11195 positron emission tomography (PK11195 PET) and evaluated age-related changes.

**Methods:**

A dynamic PK11195 PET scan was performed in 15 normal healthy adults (mean age: 29 ±8.5 years (range: 20 to 49); 7 males) and 10 children (mean age: 8.8 ±5.2 years (range: 1.2 to 17); 5 males), who were studied for potential neuroinflammation but showed no focally increased PK11195 binding. The PET images were evaluated by calculating standard uptake values and regional binding potential, based on a simplified reference region model, as well as with a voxel-wise analysis using statistical parametric mapping.

**Results:**

PK11195 uptake in the brain is relatively low, compared with the subcortical structures, and symmetrical. The overall pattern of PK11195 distribution in the brain does not change with age. PK11195 uptake was lowest in the frontal-parietal-temporal cortex and highest in the pituitary gland, midbrain, thalamus, basal ganglia, occipital cortex, hippocampus and cerebellum, in descending order. White matter showed negligible PK11195 uptake. Overall, brain PK11195 uptake increased with age, with midbrain and thalamus showing relatively higher increases with age compared with other brain regions.

**Conclusions:**

The brain shows low PK11195 uptake, which is lower in the cortex and cerebellum compared with subcortical structures, suggesting a low level of translocator protein expression. There is no hemispheric asymmetry in PK11195 uptake and the overall pattern of PK11195 distribution in the brain does not change with age. However, brain PK11195 uptake increases with age, with the thalamus and midbrain showing relatively higher increases compared with other brain regions. This increase in uptake suggests an age-related increase in translocator protein expression or the number of cells expressing these receptors or both.

## Background

The 18 kDa translocator protein (TSPO), previously known as the peripheral benzodiazepine receptor, is a hetero-oligomeric complex comprising the voltage-dependent anion channel as well as an adenine nucleotide carrier found in both the periphery and the brain [1-4]. In the normal healthy brain, TSPO is located in ependymal cells lining the ventricles, the olfactory bulb, the choroid plexus, and glial cells, including astrocytes and microglia [5,6]. Microglia are mesoderm-derived brain macrophages and represent the resident immunocompetent cells that become activated during neuroinflammation [7]. In neuroinflammation, activated microglia are the primary source of TSPO expression, with lower or insignificant contributions from astrocytes [8]. This is the rationale for *in vivo* imaging (detection) of neuroinflammation or underlying activated microglia using various radiotracers, which specifically bind to the TSPOs expressed by these activated microglia.

The carbon-11 labeled positron emission tomography (PET) tracer 1-(2-chlorophenyl)-N-methyl-N-(1-methylpropyl)-3-isoquinoline carboxamide (^11^C-[R]-PK-11195 (PK11195)) selectively binds to TSPOs and has been used most for *in vivo* imaging of neuroinflammation [9]. However, experience with this tracer in children is very limited, and detailed PK11195 brain PET kinetics and data about age-related changes are lacking. The purpose of the present study was to evaluate the brain distribution and expression of TSPOs, using PK11195 brain PET, and to evaluate the effect of age.

## Methods

### Participants

A total of 25 participants were recruited in this study: 15 normal healthy adults (mean age: 29 ± 8.5 years (range: 20 to 49); 7 males) and 10 children (mean age: 8.8 ± 5.2 years (range: 1.2 to 17); 5 males) in whom neuroinflammatory processes were being considered. Three of these children had a diagnosis of partial epilepsy, three children had encephalitis-like features without a firm diagnosis, two children had motor tics, and two had developmental delay and a history of dyskinesia. These children were selected from a group of 93 children who underwent a PK11195 PET scan due to suspected neuroinflammation. In all these children, neuroinflammation was eventually considered to be unlikely on the basis of all available clinical, radiological, serological and follow-up data, and no focal increase in PK11195 binding was detected in these children compared with the normal adult control participants. We therefore believe that this group of children likely represents a relatively normal distribution of TSPOs in the pediatric age group. All the participants had normal magnetic resonance imagining scans and none of the children were taking any steroids, benzodiazepine or anti-inflammatory drugs prior to the study.

All studies were performed in accordance with guidelines stipulated by the Human Subjects Research Committee at Wayne State University, Detroit, MI, USA, and written informed consent was obtained from adult participants and parents or guardians of the children. Oral or written assent was obtained for children age 7 to 12 years and 13 to 18 years, respectively.

### Positron emission tomography imaging procedure

The PK11195 was produced using a synthesis module, designed and built in-house [10]. PET studies were performed using the CTI/Siemens EXACT/HR whole-body positron tomograph (Siemens, Knoxville, TN, USA) after 6 hours of fasting. Prior to tracer injection, a 15-minute transmission scan of the head was acquired using rotating Ge-68 line source to correct for attenuation correction. Coinciding with the tracer injection (17 MBq/kg), a 60-minute dynamic scanning of the brain was initiated (12 × 5 minutes). Emission data were acquired in three-dimensional mode and measured attenuation correction as well as scatter and decay correction were applied to all PET images. The reconstructed image resolution was 6.5 ± 0.3 mm full-width at half maximum in-plane and 6.0 ± 0.5 mm full-width at half maximum in the axial direction. Eight children required sedation, which was performed using intravenous nembutal (3 mg/kg), as it has been reported that acute short-term use of barbiturate does not affect TSPO receptors [11].

### Positron emission tomography image processing and analysis

PET images were evaluated both qualitatively (visually) as well as quantitatively based on the calculation of standard uptake values (SUVs), reflecting overall PK11195 uptake, and binding potential (BP), reflecting specific PK11195 binding to TSPO receptors. Moreover, all images were also analyzed using a voxel-wise analysis of whole-brain PK11195 PET data using a statistical parametric approach.

#### Visual analysis

**Figure 1:**
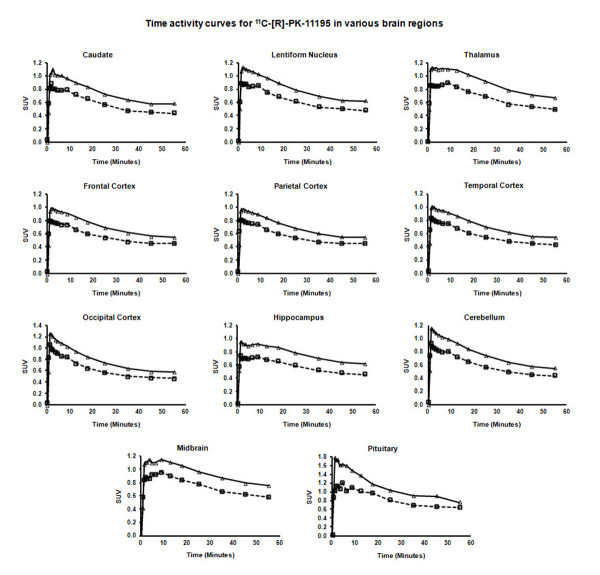
**Normalized time activity curves.** Normalized time activity curves from various brain regions showing rapid ^11^C-[R]-PK11195 uptake followed by steady washout. Triangles: adults (≥18 years of age); squares: children (<18 years of age); SUV: standard uptake value.

Initially, the dynamic image sequence was averaged between 5 and 20 minutes post injection and normalized to the injected dose per participant weight as follows: (μCi/cm^3^)/(injected activity/weight). This yieldsed semi-quantitative SUV images that were visually assessed. The time interval between 5 and 20 minutes was chosen, as this time period represented the highest tracer activity accumulation, excluding the perfusion phase (see Figure 1), and provided the best image quality.

#### Calculation of standard uptake value and binding potential

**Figure 2:**
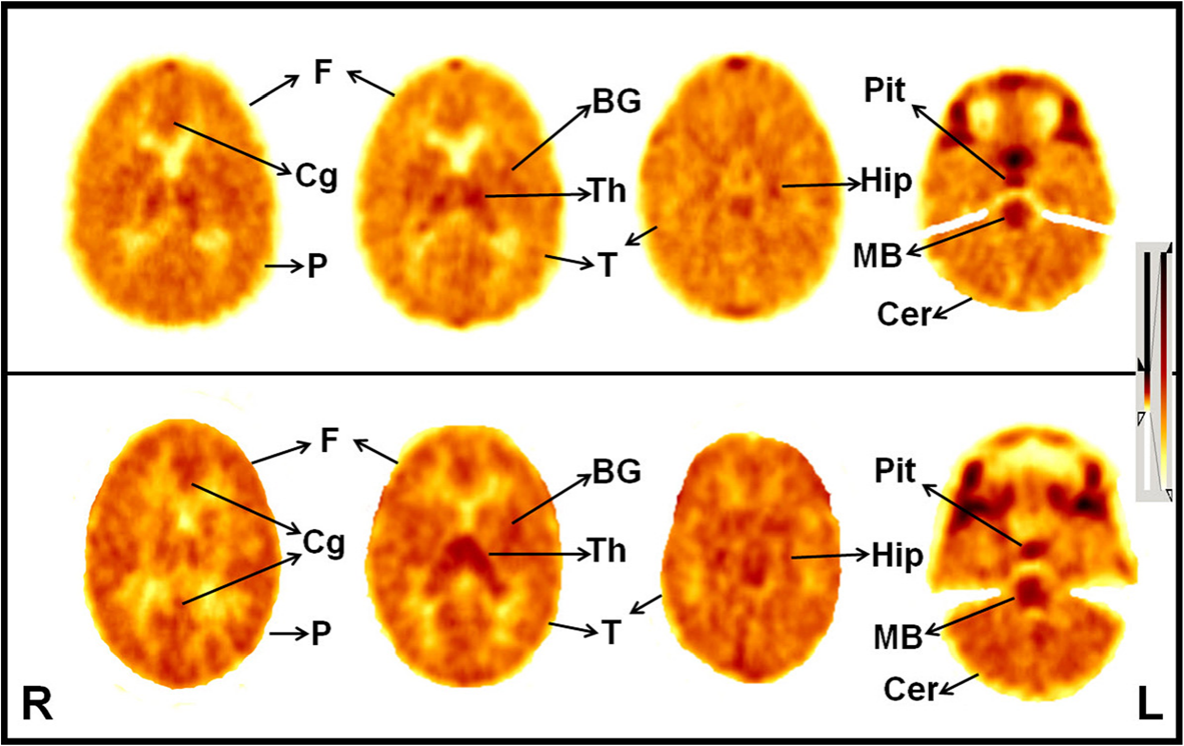
**Images of **^**11**^**C-[R]-PK11195 brain positron emissiontomography scan.** PK11195 brain PET scan showing PK11195 brain distribution in a child (upper row) and healthy normal adult (lower row). The overall brain PK11195 uptake is relatively low, suggested by much lower cortical PK11195 uptake compared to subcortical structures, and the pattern of PK11195 distribution is largely similar in both children and adults, although PK11195 uptake appears to be visually slightly higher in adults, particularly in the thalamus and midbrain. Highest PK11195 uptake is noted in the pituitary gland and midbrain, followed by thalamus, basal ganglia, occipital cortex, hippocampus and cerebellum. The rest of the cerebral cortex is showing low PK11195 uptake with minimal uptake in white matter. BG: basal ganglia; MB: midbrain; Cer: cerebellum; Cg: cingulum; F: frontal cortex; Hip: hippocampus; P: parietal cortex; Pit: pituitary gland; T: temporal cortex; Th: thalamus; R: right side; L: left side.

Regions of interest encompassing various brain regions were defined manually in the SUV images and corresponding time-activity curves (TACs) were generated from the whole dynamic sequence. To compare TACs across participants, all TACs were normalized to the injected dose per participant weight using the formula above, which numerically corresponds to the SUV values at various time points. The normalized TACs were subsequently used to calculate SUV (from 5 to 20 minutes) and the regional BP (using complete dynamic data set), based on a simplified reference tissue model [12]. In the reference tissue model, the time course of the tracer in the region of interest is expressed in terms of the tracer uptake in a reference region devoid of specific binding. It is further assumed that the level of nonspecific binding is equal in the region of interest and the reference region. The time course of the PK11195 in the region of interest C_ROI(t)_ can then be expressed as

$$C_{\mathrm{ROI}}\left(t\right)=R_{1}C_{\mathrm{ref}}\left(t\right)+\left[k_{2}-\frac{R_{1}k_{2}}{1+BP}\right]C_{\mathrm{ref}}\left(t\right)⊗\frac{-k_{2}t}{e^{1}+BP}$$

where C_ref_ (t) is the time course of tracer activity in the reference region and R1 (the ratio of delivery of the tracer between the target and reference region), k2 (the efflux rate constant from the target tissue) and BP are the parameters to be estimated using nonlinear fitting. Of specific interest for the PK11195 is the BP which characterizes the ratio between the association and dissociation constants of the ligand-receptor complex. As the brain region completely devoid of specific binding is not known with certainty, the TAC for the reference region was created using a cluster analysis approach [13]. In brief, voxels were initially constrained to gray matter and this subset of voxels was subsequently segmented into nine clusters, characterized by the maximal value and the shape of the corresponding TACs. The cluster characterized by the TACs with lowest maximal value and fastest washout was subsequently used as the reference tissue, as they were considered to be devoid of any specific binding. These clusters were found to be located in the frontal-temporal-parietal cortex, as these regions showed the lowest PK11195 uptake, both quantitatively (Figure 1 and Tables1 and 2) and qualitatively (Figure 2).

**Table 1:** **Standard uptake value of **^**11**^**C-[R]-PK11195 in various brain regions**

		**SUV**_**5 to 20 minutes **_**(mean ± SD (95%CI))**		
	**Side**	**Adults (n = 15)**	**Children (n = 10)**	***P***
**Pituitary gland**		1.04 ±0.19 (1.27 to 1.53)	1.01 ±0.43 (0.60 to 2.09)^a^	0.97
**Midbrain**		1.11 ±0.17 (1.01 to 1.2)	0.9 ±0.21 (0.75 to 1.01)	0.001
**Thalamus**	Left	1.09 ±0.14 (1.01 to 1.17)	0.84 ±0.23 (0.67 to 1)	0.002
	Right	1.08 ±0.12 (1.02 to 1.15)	0.84 ±0.24 (0.67 to 1.01)	0.002
**Lentiform nucleus**	Left	1.0 ±0.12 (0.94 to 1.07)	0.78 ±0.19 (0.65 to 0.92)	0.002
	Right	0.97 ±0.15 (0.89 to 1.05)	0.76 ±0.2 (0.62 to 0.91)	0.006
**Caudate**	Left	0.93 ±0.11 (0.87 to 0.99)	0.73 ±0.17 (0.61 to 0.85)	0.001
	Right	0.92 ±0.11 (0.86 to 0.99)	0.73 ±0.17 (0.60 to 0.85)	0.002
**Hippocampus**	Left	0.88 ±0.11 (0.82 to 0.94)	0.69 ±0.17 (0.57 to 0.81)	0.002
	Right	0.87 ±0.11 (0.81 to 0.93)	0.69 ±0.16 (0.57 to 0.81)	0.002
**Cerebellum**	Left	0.97 ±0.11 (0.91 to 1.03)	0.77 ±0.2 (0.63 to 0.91)	0.003
	Right	0.99 ±0.12 (0.92 to 1.05)	0.76 ±0.20 (0.62 to 0.90)	0.001
**Frontal cortex**	Left	0.95 ±0.11 (0.88 to 1.01)	0.73 ±0.17 (0.61 to 0.86)	0.001
	Right	0.94 ±0.11 (0.88 to 1.00)	0.73 ±0.17 (0.61 to 0.85)	0.001
**Parietal cortex**	Left	0.87 ±0.11 (0.81 to 0.93)	0.68 ±0.17 (0.56 to 0.8)	0.002
	Right	0.87 ±0.11 (0.81 to 0.93)	0.68 ±0.17 (0.56 to 0.8)	0.002
**Temporal cortex**	Left	0.86 ±0.10 (0.80 to 0.92)	0.68 ±0.17 (0.55 to 0.8)	0.002
	Right	0.85 ±0.11 (0.79 to 0.91)	0.68 ±0.16 (0.56 to 0.8)	0.005
**Occipital cortex**	Left	0.91 ±0.11 (0.85 to 0.98)	0.68 ±0.19 (0.54 to 0.82)	0.001
	Right	0.88 ±0.09 (0.82 to 0.93)	0.69 ±0.19 (0.55 to 0.82)	0.003

^a^Pituitary SUV values available in three children only, as it could not be estimated in seven children because of an inability to reliably delineate the pituitary from the surrounding lymphoid tissue/circular venous plexus in these children. *CI* confidence intervals; *SD* standard deviation; *SUV* standard uptake value.

**Table 2:** **Binding potential values of **^**11**^**C-[R]-PK11195 in various brain regions**

		**Binding potential (mean ±SD (95% CI))**		
	**Side**	**Adults (n = 15)**	**Children (n = 10)**	***P***
**Pituitary gland**		0.62 ±0.18 (0.49 to 0.75)	0.61 ±0.19 (0.15 to 1.07)*	0.95
**Midbrain**		0.45 ±0.09 (0.38 to 0.48)	0.41 ±0.08 (0.40 to 0.48)	0.09
**Thalamus**	Left	0.35 ±0.04 (0.33 to 0.37)	0.28 ±0.13 (0.19 to 0.37)	0.08
	Right	0.34 ±0.07 (0.30 to 0.37)	0.29 ±0.12 (0.21 to 0.37)	0.20
**Lentiform nucleus**	Left	0.20 ±0.05 (0.17 to 0.23)	0.19 ±0.07 (0.14 to 0.24)	0.63
	Right	0.15 ±0.05 (0.12 to 0.18)	0.15 ±0.07 (0.09 to 0.21)	0.92
**Caudate**	Left	0.10 ±0.05 (0.07 to 0.13)	0.08 ±0.06 (0.03 to 0.13)	0.45
	Right	0.09 ±0.04 (0.07 to 0.12)	0.08 ±0.05 (0.01 to 0.14)	0.46
**Hippocampus**	Left	0.11 ±0.04 (0.07 to 0.14)	0.07 ±0.04 (0.03 to 0.12)	0.20
	Right	0.10 ±0.06 (0.06 to 0.14)	0.10 ±0.06 (0.04 to 0.15)	0.83
**Cerebellum**	Left	0.10 ±0.04 (0.07 to 0.12)	0.08 ±0.03 (0.05 to 0.11)	0.45
	Right	0.09 ±0.04 (0.07 to 0.11)	0.07 ±0.03 (0.05 to 0.10)	0.24
**Frontal cortex**	Left	0.06 ±0.03 (0.04 to 0.07)	0.05 ±0.01 (0.04 to 0.06)	0.47
	Right	0.05 ±0.03 (0.03 to 0.07)	0.05 ±0.03 (0.03 to 0.07)	0.85
**Parietal cortex**	Left	0.04 ±0.03 (0.02 to 0.05)	0.03 ±0.02 (0.01 to 0.04)	0.64
	Right	0.04 ±0.01 (0.03 to 0.05)	0.04 ±0.03 (0.02 to 0.06)	0.53
**Temporal cortex**	Left	0.05 ±0.03 (0.03 to 0.07)	0.05 ±0.02 (0.03 to 0.07)	0.85
	Right	0.05 ±0.02 (0.03 to 0.06)	0.02 ±0.02 (0.03 to 0.07)	0.91
**Occipital cortex**	Left	0.11 ±0.05 (0.08 to 0.14)	0.11 ±0.04 (0.08 to 0.13)	0.79
	Right	0.12 ±0.03 (0.10 to 0.14)	0.12 ±0.02 (0.10 to 0.13)	0.88

*Pituitary binding potential values available in three children only, as it could not be estimated in seven children because of an inability to reliably delineate the pituitary from the surrounding lymphoid tissue/circular venous plexus in these children. *CI* confidence intervals; *SD* standard deviation.

#### Voxel-wise analysis (statistical parametric mapping)

Voxel-wise analysis of SUV images was performed to assess the global differences in PK11195 uptake between adults (≥18 years of age; n = 15) and children (6 to 18 years of age; n = 8), as we have previously shown the feasibility of statistical parametric mapping (SPM) analysis using an adult template [14]. The analysis was performed using SPM8 software package (Wellcome Department of Cognitive Neurology, Institute of Neurology, London, UK). Spatial normalization was performed using (7 × 8 × 7) number of basis functions with medium regularization, 12 nonlinear iterations and a smoothing kernel of 16 mm, resulting in an image resolution of approximately 18 mm full-width at half maximum. Subsequently, to analyze regionally specific effects, a general linear model was used to assess differences among parameter estimates (specified by contrasts) and the significance of individual contrasts was then tested using the SPM(t) statistics for each voxel. The confounding effect of global activity was removed using proportional scaling and a two-sample t-test was applied, with the resulting SPM(t) thresholded at *P* <0.05 (corrected for family-wise error) with a cluster threshold of 20 voxels.

### Statistical analysis

Values are expressed as mean ±SD. The Spearman’s correlation analysis was performed to assess the correlation of age with BP or SUV and a repeated measure analysis of variance (ANOVA) was performed to assess hemispheric asymmetry. A mixed-design ANOVA was performed to evaluate any difference between children (<18 years of age) and adults (≥18 years of age) with respect to side (left/right) and brain region, without including the pituitary, which was analyzed separately. This was followed by a simple effect test to compare the difference in any specific brain region. A *P*-value less than 0.05 was considered significant. The SPSS program (version 18.0; SPSS Inc.) was used for the statistical analyses.

## Results

### Visual analysis

SUV images of PK11195 in the brain of a child and an adult control are shown in Figure 2. The overall brain PK11195 uptake was relatively low, suggested by much lower cortical PK11195 uptake compared with subcortical structures as evidenced by both quantitative (Tables1 and 2) and qualitative (visual; Figure 2) data. The pattern of PK11195 distribution was similar in both children and adults, although PK11195 uptake appeared to be visually slightly higher in adults. Visual assessment showed highest PK11195 uptake in the pituitary gland and midbrain, followed by thalamus, basal ganglia, occipital cortex, hippocampus and cerebellum. By contrast, the rest of the cerebral cortex showed low PK11195 uptake with minimal uptake in white matter.

### Standard uptake values analysis

**Figure 3:**
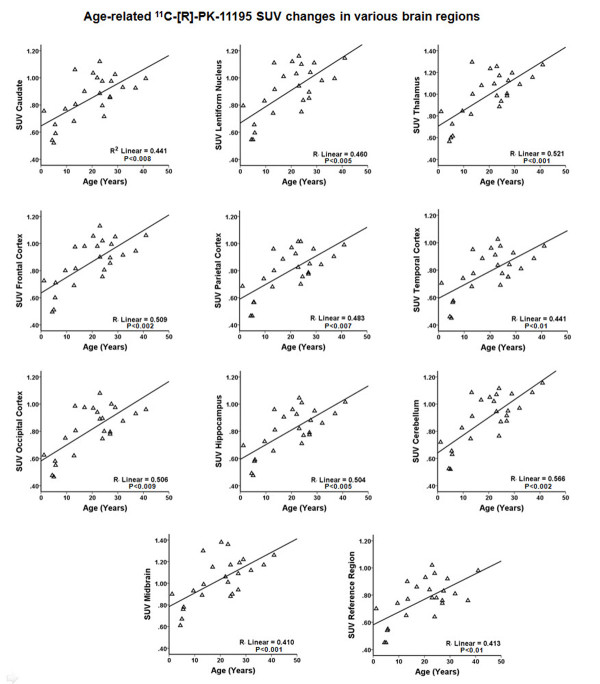
**Effect of age on brain **^**11**^**C-[R]-PK11195 uptake.** Correlation analysis between age and PK11195 uptake (standardized uptake value; SUV) showed a significant age-related increase in brain PK11195 uptake.

Normalized TACs (for injected dose and participant weight) for most brain regions showed rapid increase of tracer, reaching a peak within 1 to 2 minutes of tracer injection followed by a steady washout, with TACs being lower in children compared with adults (Figure 1). Consistent with the visual appearance, the highest tracer uptake (SUV) was observed in the pituitary gland followed by the midbrain, thalamus, basal ganglia, hippocampus and cerebellum, with the lowest uptake seen in the frontal, parietal and temporal cortices (Table 1). The occipital cortex displayed the highest SUV among cortical structures; however, this region also showed the most rapid washout. No left-right asymmetry was observed for SUV values. SUVs for all brain regions showed a very significant positive correlation with age (Figure 3) and were found to be significantly higher in adults than in children (Table 1).

### Binding potential analysis

BP analyses were consistent with visual and SUV analyses, with the highest BP being determined in the pituitary gland and midbrain followed by thalamus and basal ganglia, and the lowest BP in cortical areas (Table 2). PK11195 binding in the cerebellum was found to be higher than that in the frontal-parietal-temporal cortex. No left-right asymmetry was detected for any of the tested brain regions. However, unlike the SUVs, there was no significant correlation between BP values and age, except for thalamus which showed a trend (*P* = 0.09).

### Voxel-wise analysis

**Figure 4:**
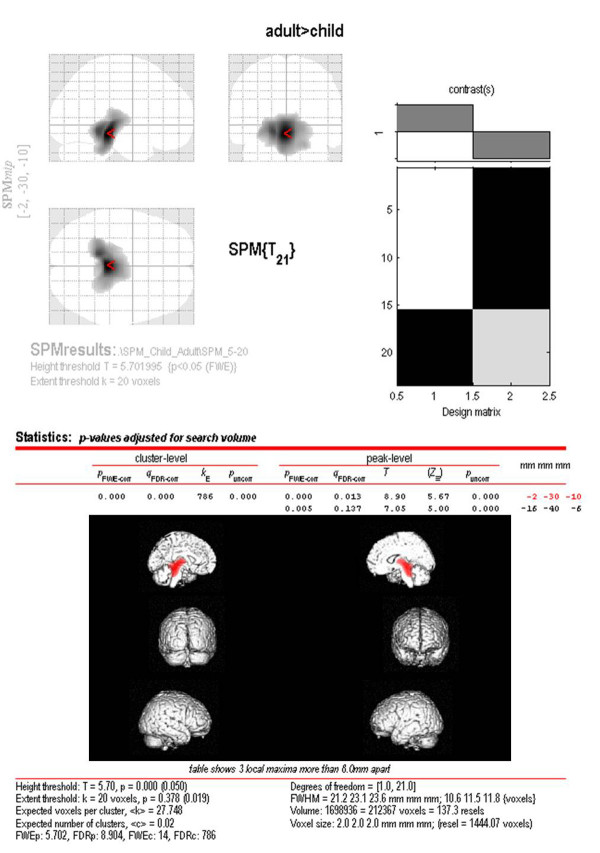
**Result of voxel-based analysis of **^**11**^**C-[R]-PK11195 positron emission tomography scans.** Voxel-wise statistical parametric mapping analysis of PK11195 standard uptake value images, carried out between adults and children, showed significantly higher PK11195 uptake in adults in the thalamus and midbrain region only.

SPM analysis of SUV images, carried out between adult and children, showed significantly higher PK11195 SUV in adults in the region of the thalamus and the midbrain only (*P* = 0.001, corrected), suggesting a relatively higher age-related increase in thalamic and midbrain PK11195 SUV compared with other brain regions (Figure 4). There were no statistically significant differences determined for the cortex, basal ganglia or cerebellum.

## Discussion

The results of our study show that brain PK11195 uptake is low with the overall pattern of PK11195 brain distribution not changing with age. The PK11195 uptake was found to be lowest in the frontal-parietal-temporal cortex and highest in the pituitary gland, midbrain and thalamus. Intermediate tracer uptake was determined in the basal ganglia, occipital cortex, hippocampus and cerebellum, in descending order. We did not observe any hemispheric asymmetry in PK11195 uptake. However, overall PK11195 uptake increased with age, with the thalamus and midbrain showing relatively higher increases compared with other brain regions. Interestingly, we did not find similar significant age-related changes in BP values, except for the thalamus, which showed a trend. The differences in age-related changes between the SUV and BP may be because BP calculation depended on reference region uptake values, and a generalized whole-brain increase in PK11195 uptake, including the ‘reference region’ (See Figure 3), might have rendered BP values largely unchanged, except for the thalamus, where a relatively higher increase in PK11195 uptake resulted in a trend toward statistical significance.

Our findings indicate that, in the brain, there is a relatively low level of TSPO expression, which increases with age, probably due to an increase in TSPO number and/or expression, an increase in cells containing these receptors, or both. Moreover, TSPO expression is lowest in the cortex, particularly in the frontal, parietal and temporal cortices, and highest in the pituitary gland and midbrain, followed by thalamus, basal ganglia, hippocampus and cerebellum. Most importantly, the TSPO distribution pattern remains unchanged with increasing age, being largely similar in children and adults, and no hemispheric asymmetry is seen in the pattern or level of TSPO expression.

The observed PK11195 binding pattern in the present study is consistent with the normal brain TSPO distribution described in the literature [5,6,15]. Age-related increases in microglia and upregulation of TSPOs have been reported in several animal, human and postmortem studies [16-23]. It appears that aging may serve as a priming stimulus for microglia [24], and ontogenic changes in TSPOs, related to increase in glial cells, have been reported in rat and guinea pig brain [25-27]. Significantly greater numbers of microglia and astrocytes were reported in the hippocampus of aged female mice [28], and an age-related increase in the expression of a microglia-associated antigen was reported in rat and monkey brain [29,30]. In a human postmortem study, an age-related increase in the number of enlarged and especially phagocytic microglia was found in the brain of neurologically normal individuals, aged 2 to 80 years [31]. It appears that these age-related changes may be due to chronic and persistent neuronal damage over the years, shown to occur in the brains of experimental animals [32]. Age-related oxidative damage to DNA, more for mitochondrial than for nuclear DNA [33], and accumulation of glycated proteins occurs in normal aging human brain [34]. These changes lead to microglial activation, which is considered to be instrumental in the removal of such damage and routine ‘house-cleaning’. Therefore, an age-related increase in brain PK11195 uptake as seen in the present study is likely related to the age-related increase in activated microglia and increased TSPO expression.

We also found that the age-related increase in PK11195 uptake was higher in the thalamus and midbrain compared with other cortical and subcortical regions, which is consistent with a finding reported previously in an older adult group [35]. In a study of normal adults aged 32 to 80 years, Cagnin *et al*. found that regional PK11195 BP did not significantly change with age, except in the thalamus, which showed an age-dependent increase [35]. The thalamus is connected to widespread cortical regions. Similarly, the midbrain is reciprocally connected to several brain regions, primarily the thalamus and basal ganglia, and has one of the highest densities of microglia, particularly in the substantia nigra [36]. Any subtle injury in various brain regions, as seen with normal aging, may induce a similar but cumulative and amplified microglial response in the thalamus and midbrain, leading to increased number of TSPOs and therefore increased PK11195 binding [37,38]. This activation may facilitate remodeling, which is an adaptive process in long-term plasticity in response to progressive age-related neuronal loss. Further synaptic reorganization, most likely a compensatory response to the decline in age-related brain function associated with the reduction in functional integration across the distributed neuronal network (including the thalamus and midbrain), may induce progressively increased microglial activation and proliferation. It is interesting to note that enhanced age-related microglial activation in the midbrain, triggered by various insults and leading to inflammation-derived oxidative stress and cytokine-dependent toxicity, may contribute to nigrostriatal pathway degeneration and hasten progression of disease in idiopathic Parkinson’s disease [39]. Indeed, markedly elevated neuroinflammation has been reported in the pons along with several other brain regions in patients with idiopathic Parkinson’s disease compared with age-matched healthy controls [40]. Therefore, our findings of a higher age-related increase in PK11195 uptake in the midbrain may be reflective of an age-related increase in microglial activation that, beyond a threshold, depending upon the type and severity of noxious stimuli or insults, may result in some neurological impairment. However, there may be other possible explanations that need to be elucidated and explored in future studies.

A few adult studies have reported PK11195 BP values in several brain regions based on similar methodology, and our values are largely consistent with these studies [35,41]. However, no study is available regarding the pediatric population, and detailed PK11195 brain PET kinetics and data about age-related changes is lacking. We believe that our study fills the existing knowledge gap and provides important information about PK11195 brain kinetics in the maturing brain and age-related changes in TSPO distribution. Our data will also assist in the interpretation and analysis of PK11195 PET scans in various neuroinflammatory conditions, especially in the pediatric population. The present study also suggests that SPM analysis can be useful for the evaluation of global cortical differences in PK11195 uptake.

### Limitations

One of the limitations of this work is the selection of a reference region for the calculation of BP, and as a result the calculated BP values may not be the true estimate of actual receptor ligand binding. Ideally, arterial sampling should be performed to obtain the true input function; however, it is not practical in routine clinical work, particularly in the pediatric population. A cluster analysis may be a reasonable compromise to obtain an estimate of a reference region as a substitute for the true input function, but this approach is only valid in the case where the whole brain is not expected to be uniformly involved in the neuroinflammatory process. Overall visual and SUV analysis may help in these cases.

Another limitation is the selection of children; although we recruited only those children in whom focal neuroinflammation was ruled out, they were not completely healthy children. It is difficult to recruit completely ‘normal’ children because of ethical constraints in exposing children to the radiation associated with PET studies. Further, allowing for the fact that a few of our children might have had subtle neuroinflammation or some other conditions which might have led to increased PK11195 uptake, completely normal children would be expected to have even lower PK11195 uptake values than observed here, and therefore will further enhance and strengthen our finding of age-related increases in brain PK11195 uptake. It is worth noting, however, that age-related changes observed in the pediatric age-range may not be completely representative due to possible differences in the level of neuroinflammation in these children, if any. It also infers that the normal pediatric PK11195 values should be equal to or less than the normal adult values and, as a result, pediatric PK11195 values that exceed adult values can be safely considered to be abnormal. This information has a practical implication for future studies using PK11195 PET in children, where only normal adult PK11195 data may be available for comparison purpose.

## Conclusions

The brain shows low PK11195 uptake, lower in the cortex and cerebellum compared with subcortical structures, suggesting low level of TSPO expression. There is no hemispheric asymmetry in PK11195 uptake and the overall pattern of PK11195 distribution in the brain does not change with age. However, brain PK11195 uptake increases with age, with the thalamus and midbrain showing relatively higher increases compared with other brain regions, suggesting an age-related increase in TSPO expression or the number of cells expressing these receptors, or both.

## Competing interests

The authors declare that they have no competing interests.

## Authors’ contributions

AK was involved in the design of the study, data acquisition, data analysis and manuscript preparation. OM was involved in the data analysis and manuscript preparation. VS was involved in the data analysis and manuscript preparation. DC was involved in study design, data analysis and manuscript preparation. PC was involved in data acquisition and manuscript preparation. HC was involved in study design, data acquisition and manuscript preparation. All authors read and approved the final manuscript.
